# Research on nonlinear dynamic characteristics of high-speed gear in two-speed transmission system

**DOI:** 10.1038/s41598-023-47981-1

**Published:** 2023-11-24

**Authors:** Wuzhong Tan, Jiangming Wu, Zhihui Liu, Xueshen Wu, Jiahao Zhang

**Affiliations:** 1 School of Mechanical and Electrical Engineering, Central South University, Changsha, 410083 China; 2 AECC Hunan Aviation Powerplant Research Institute, Zhuzhou, 412002 China; 3 School of Mechanical and Energy Engineering, Shaoyang University, Shaoyang, 422000 China; 4 Aviation Military Representative Office of the PLA Army Equipment Department, Zhuzhou, 412002 China

**Keywords:** Engineering, Mathematics and computing

## Abstract

The working performance and service life of the two-speed transmission system directly affects the performance and service life of helicopters and other equipment. One of the main tasks of the two-speed transmission system research is to improve its dynamic characteristics. For the two-speed transmission system in high-speed gear, a purely torsional nonlinear dynamic differential equation set considering the number of planetary gears, backlash, and clutch dynamic load is established by using the lumped parameter method, and the equations are dimensionless. Then the dimensionless differential equation set is solved by using the variable step-size fourth-order Runge–Kutta method, and the phase diagram and Poincare diagram of high-speed gear are obtained. By changing the dynamic friction coefficient of the friction clutch and the backlash of the gear pair, the influence of parameter change on the nonlinear dynamic characteristics of the system is analyzed. The results show that, with the increase of excitation frequency, the system has experienced single cycle, quasi-cycle, chaos, and double cycle, then changed from double cycle to chaotic motion, and then changed from chaotic motion to double cycle and single cycle motion in turn, and found the path to chaos. In the low-frequency band, reducing the friction coefficient of the friction clutch can reduce vibration amplitude; In the middle-frequency band, reducing the friction coefficient will make the system tend to unstable vibration. In the high-frequency band, it is a single-cycle movement, which is not affected by friction coefficient.

## Introduction

The two-speed transmission system is a variable speed system with two speed outputs, which is widely used in the fields of helicopter, tank, loader, automobile and so on. A two-speed transmission system is added in the main subtraction of the helicopter, which can realize helicopter cruise and hover (start and stop) and achieve the lowest fuel consumption. The typical structure of two-speed transmission system consists of friction clutch, overrunning clutch and planetary gear transmission system^1–3^. The performance and service life of the two-speed transmission system directly affect the performance and service life of the helicopter and other equipment. The study of nonlinear characteristics of two-speed transmission system can guide the optimal design and manufacture of transmission system.

At present, the friction clutch and the planetary gear transmission system have been widely used, and the research on the nonlinear characteristics of the coupling transmission system between the friction clutch and the planetary gear has been a hotspot. Han^4^ established a nonlinear dynamics model considering time-varying meshing stiffness, tooth side clearance and static transmission error, and solved it by using numerical integration method, and analyzed the dynamics characteristics of the system through global bifurcation diagram. Li^5^ studied the meshing characteristics of planetary gear train when the position of the planetary frame changes under variable loads. Wang^6^ established a torsional dynamics model of planetary gear transmission system considering friction, time-varying meshing stiffness, meshing damping and clearance, solved the system vibration equation by Runge–Kutta method, and analyzed the bifurcation and chaos characteristics of the system through bifurcation diagrams and phase diagrams. Luo^7^ established a dynamic model including time-varying meshing stiffness, sliding friction and torque, and studied the influence of sliding friction on the dynamic characteristics of planetary gear mechanisms. For the 3K-ii planetary gear system, Sang^8^ established a torsional vibration dynamic model of the system by using the lumped parameter method, and analyzed the influence of tooth root crack on the system. Ryali^9^ studied the load distribution and dynamic characteristics of planetary gear system under internal and external excitation. Xu^10^ established a new gear tooth modification model according to the characteristics of tooth top modification and tooth profile modification of planetary gear train. Ling^11^ analyzed the motion and various nonlinear dynamics characteristics of planetary gear system by using global bifurcation diagram, FFT spectrum, Poincare diagram, phase diagram and maximum Lyapunov index. Li^12^ established the reliability prediction model of helicopter planetary gear train under partial load. Xiang^13^ identified the influence of system motion on the change of backlash by using global bifurcation diagram, maximum Lyapunov index (LLE), FFT spectrum, Poincare diagram, phase diagram and time series.

The research on nonlinear characteristics of planetary gear transmission system mainly focuses on modeling methods, solving methods, stability judgment and other aspects. The models mainly include pure torsional model and bending-torsional coupling model, and the time-varying meshing stiffness, tooth clearance and comprehensive meshing error are usually considered in the modeling. The nonlinear dynamics of planetary gear transmission system can be solved by analytical method and numerical method.

The dynamics of friction clutch is mainly studied by modeling and analyzing the clutch independently or simplifying other mechanical structures. The analysis and solution methods are mainly numerical iteration, concentrated parameters and finite element method. Li^14^ analyzed the self-excited vibration characteristics of clutch and discussed the influence of clutch related physical parameters on its performance based on the established 4-DOF nonlinear multi-body dynamics model and Karnopp friction model. Bao^15,16^ established the transient thermal analysis model of friction clutch and the motion coupling model in the engagement process, and studied the influence of the groove shape of the friction disc on the transient temperature field in the clutch engagement process and the influence of relevant parameters on the speed and transmission torque of clutch engagement. Wang^17^ proposes an improved model for calculating the meshing stiffness of a helical gear system caused by a gear crack, which takes into account the transverse and axial effects of the gear tooth stiffness and the gear foundation stiffness. The results show that the meshing stiffness of the gear is greatly reduced by the existence of cracks. The time domain vibration response of cracked gear is sudden, and the frequency spectrum shows that the more serious the crack, the more abundant the side frequency component and the higher the amplitude. Wang^18^ proposed an improved meshing stiffness calculation model for helical gear pairs, which fully considered not only the tooth stiffness of axial gear and the foundation stiffness of axial gear, but also the transverse gear tooth stiffness and foundation stiffness affected by surface roughness under elastohydrodynamic lubrication. The results show that compared with the traditional method, the improved meshing stiffness calculation model can obtain the meshing stiffness under actual lubrication conditions, but the traditional method ignores the axial meshing force and the friction force acting on the gear teeth and gear base.

Compared with the single clutch or planetary gear, the dynamic characteristics of the coupling system composed of friction clutch and planetary gear are more complex. Although this kind of system is widely used, the nonlinear characteristics of friction clutch-planetary gear system are rarely studied. Aleksandar^19^ established a simulation model of friction clutch and planetary gear train, and conducted a simulation analysis on the transition process of planetary gear train in the shift process. Michel Bauer^20^ established a friction clutch-planetary gear train model suitable for hybrid power and conducted a simulation study on its shifting characteristics. Wang^21^ established a planetary gear torsional vibration model considering clutch friction torque and other factors, and studied the influence of clutch friction torque and planetary gear meshing stiffness on system vibration. Chen^22^ established a nonlinear dynamics model of two-stage gear transmission system with overrunning clutch, numerically solved the model with Runge–Kutta method, and studied the influence of gear modulus and clutch torsional stiffness on dynamic characteristics of the system.

Through the above analysis, it can be seen that although there have been some studies on the nonlinear characteristics of planetary gear and friction clutch, there are still few studies on the coupling system combined with them, and the influence of the dynamic friction coefficient of friction clutch on the stability of the coupling system is not clear in the research process. Therefore, this paper intends to establish pure torsional nonlinear dynamic differential equations considering the number of planetary wheels, gear clearance and clutch dynamic load by using the lumped-parameter method, and then solve the dimensionless differential equations by using the four-order Runge–Kutta method with variable step size. The influence of dynamic friction coefficient of friction clutch on nonlinear dynamic characteristics of two-speed transmission system is analyzed qualitatively. The research results can provide reference for the optimization design and manufacturing of the subsequent system.

## High-gear in-gear dynamics model of a two-speed transmission system

**Figure 1:**
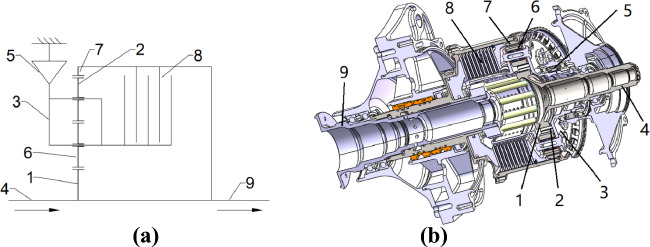
A two-speed drivetrain: (**a**) Schematic with (1) The sun wheel, (2) The first stage planetary wheel, (3) The planetary shelf, (4) Input axis, (5) Overrunning clutch, (6) The first Second planetary wheel, (7) Inner gear ring, (8) Friction clutch, and (9) Output shaft; (**b**) 3D model.

**Figure 2:**
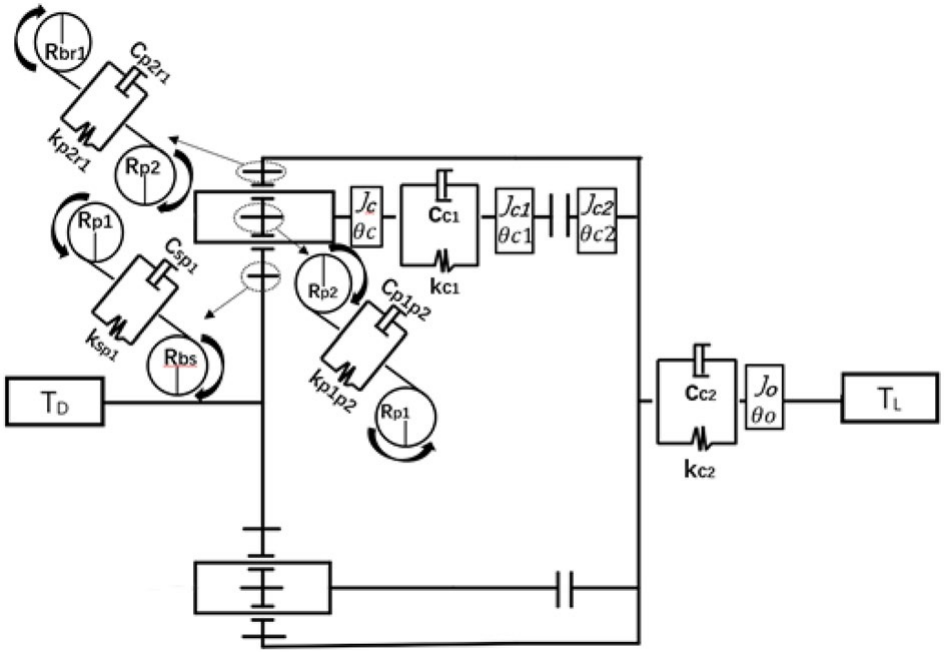
High-speed gear dynamics model of two-speed transmission system.

The structure schematic diagram of the two-speed transmission system is shown in Fig. 1. When the friction clutch is released, the planetary frame is connected with the overrunning clutch in the locked state. The power goes through the sun wheel in turn, the middle two planetary wheels, and then the inner gear ring output. At this time, it corresponds to the low gear state. When the friction clutch is engaged, the overrunning clutch is in the overrunning state, and the planetary frame can rotate freely at this time. The power also goes through the sun wheel, the middle two planetary wheels, and the inner gear ring output, which corresponds to the high gear state at this time. In this paper, the nonlinear characteristics of two-speed transmission system are studied for high speed in gear. Its dynamic model is shown in Fig. 2.

In the Fig. 2, *R*_*bs*_ is the radius of the solar wheel base circle;* R*_*bp*1_ is the radius of the base circle of the first stage planetary gear.* R*_*bp*2_ is the radius of the base circle of the second planetary gear.* R*_*br*1_ is the radius of the base circle of the inner gear ring;* T*_*D*_ is the driving torque;* T*_*L*_ is the load moment;* J*_*C*_ is the moment of inertia of the planetary frame; *θ*_*c*_ is the torsional deformation of the planetary frame;* J*_*C*1_is the moment of inertia of the friction clutch input end;* θ*_*c*1_ is the torsional deformation of the input end of the friction clutch;* J*_*C*2_ is the rotational inertia of the output end of the friction clutch;* θ*_*c*2_ is the torsional deformation of the output end of the friction clutch;* J*_*o*_ is the moment of inertia of the load of the two-speed transmission system; *θ*_*o*_ is the torsional deformation of the load shaft of the two-speed transmission system.* K*_*sp*1_ is the time-varying meshing stiffness of the solar wheel and the first stage planetary wheel;* C*_*sp*1_ is the meshing damping of the sun wheel and the first stage planetary wheel;* K*_*p*1*p*2_ is the time-varying meshing stiffness of the second stage planetary gear and the first stage planetary gear.* C*_*p*1*p*2_ is the meshing damping between the second stage planetary wheel and the first stage planetary wheel;* K*_*p*2*r*1_ is the time-varying meshing stiffness between the inner gear ring and the second stage planetary gear.* C*_*p*2*r*1_ is the meshing damping of the inner gear ring and the second stage planetary gear.

## A mathematical model of a two-speed transmission system in high gear

As shown in Fig. 2, *K*_*sp*1_(*t*)is the time-varying meshing stiffness of the solar wheel and the first stage planetary gear pair. Its value can be regarded as a rectangular wave, as shown in Eqs. (1)–(4):

1 $$k_{sp1} (t) = k_{sp1} (t + 2\pi /\omega ) = k_{sp1} + \sum\limits_{r = 1}^{R} {k_{sp1r} \cos (r\omega_{e} t{ - }\varphi_{r} )} ,$$

2 $${{k_{sp1} }}/ {{k_{tp} }} = \epsilon_{sp1} ,$$

3 $${{k_{\text{sp1r}} }}/{{k_{tp} }} = {{\sqrt {2 - 2\cos (2\pi r(\epsilon_{sp1} - 1))} }} / {(\pi r)},$$

4 $$\varphi_{{\text{sp}}1r} = a\tan ({{(1 - \cos (2\pi r(\epsilon_{sp1} - 1)))}} / {(\sin (2\pi r(\epsilon_{sp1} - 1)))}),$$

5 $$\left\{\begin{array}{c}{J}_{S}{\ddot{\theta }}_{S}+\sum_{i=1}^{N}({C}_{sp1i}({R}_{bs}{\dot{\theta }}_{s}-{R}_{bp1i}{\dot{\theta }}_{p1i}-{R}_{bc}{\dot{\theta }}_{c}-{\dot{e}}_{sp1i}\left(t\right)){R}_{bs}+{K}_{sp1i}({R}_{bs}{\theta }_{s}\\ -{R}_{bp1i}{\theta }_{p1i}-{R}_{bc}{\theta }_{c}-{e}_{sp1i}(t)){R}_{bs})={T}_{s}\\ {J}_{p1i}{\ddot{\theta }}_{p1i}+\frac{{R}_{bp1i}}{{R}_{bc}}{J}_{p1i}{\ddot{\theta }}_{c}-{C}_{sp1i}({R}_{bs}{\dot{\theta }}_{s}-{R}_{bp1i}{\dot{\theta }}_{p1i}-{R}_{bc}{\dot{\theta }}_{c}-{\dot{e}}_{sp1i}\left(t\right)){R}_{p1i}\\ -{K}_{sp1i}(({R}_{bs}{\theta }_{s}-{R}_{bp1i}{\theta }_{p1i}-{R}_{bc}{\theta }_{c}-{e}_{sp1i}(t)){R}_{p1i}\\ +{K}_{p1ip2i}({R}_{bp1i}{\theta }_{p1i}+{R}_{p2i}{\theta }_{p2i}-{e}_{p1ip2i}\left(t\right)){R}_{p1i}+{C}_{p1ip2i}({R}_{p1i}{\dot{\theta }}_{p1i}\\ +{R}_{bp2i}{\dot{\theta }}_{p2i}-{\dot{e}}_{p1ip2i}\left(t\right)){R}_{p1i}=0\\ {J}_{p2i}{\ddot{\theta }}_{p2i}+\frac{{r}_{bp2i}}{{r}_{bc}}{J}_{p2i}{\ddot{\theta }}_{c}-{C}_{p1ip2i}({R}_{bp1i}{\dot{\theta }}_{p1i}+{R}_{bp2i}{\dot{\theta }}_{p2i}-{\dot{e}}_{p1ip2i}\left(t\right)){R}_{p2i}\\ -{K}_{p1ip2i}(({R}_{bp1i}{\theta }_{p1i}+{R}_{bp2i}{\theta }_{p2i}-{e}_{p1ip2i}\left(t\right)){R}_{p2i}+{C}_{p2ir1}({R}_{br1}{\dot{\theta }}_{r1}\\ -{R}_{bp2i}{\dot{\theta }}_{p2i}-{R}_{bc}{\dot{\theta }}_{c}-{\dot{e}}_{p2ir1}\left(t\right)){R}_{p2i}+{K}_{p2ir1}({R}_{br1}{\theta }_{r1}-{R}_{bp2i}{\theta }_{p2i}\\ -{R}_{bc}{\theta }_{c}-{e}_{p2ir1}\left(t\right)){R}_{p2i}=0\\ {J}_{r1}{\ddot{\theta }}_{r1}+\sum_{i=1}^{N}\left({C}_{p2ir1}({R}_{br1}{\dot{\theta }}_{r1}-{R}_{bp2i}{\dot{\theta }}_{p2i}-{R}_{bc}{\dot{\theta }}_{c}-{\dot{e}}_{p2ir1}\left(t\right)){R}_{r1}\right){R}_{r1}+{K}_{p2ir1}({R}_{br1}{\theta }_{r1 } \\ -{R}_{bp2i}{\theta }_{p2i}-{R}_{bc}{\theta }_{c}-{e}_{p2ir1}\left(t\right)){R}_{r1})={T}_{r1}\\ \left({J}_{c}+{NJ}_{p1i}+{NJ}_{p2i}\right){\ddot{\theta }}_{c}+\sum_{i=1}^{N}(\frac{{r}_{bc}}{{r}_{bp1i}}{J}_{p1i}{\ddot{\theta }}_{p1i}+\frac{{r}_{bc}}{{r}_{p2i}}{J}_{p2i}{\ddot{\theta }}_{p2i})\\ -\sum_{i=1}^{N}({K}_{sp1i}{\theta }_{sp1i}({R}_{bs}{\theta }_{s}-{R}_{bp1i}{\theta }_{p1i}-{R}_{bc}{\theta }_{c}-{e}_{sp1i}(t){R}_{bs}\\ +{K}_{p2ir1}({R}_{br1}{\theta }_{r1}-{R}_{bp2i}{\theta }_{p2i}-{R}_{bc}{\theta }_{c}-{e}_{p2ir1}\left(t\right)){R}_{bs})\\ -\sum_{i=1}^{N}({C}_{sp1i}({R}_{bs}{\dot{\theta }}_{s}-{R}_{bp1i}{\dot{\theta }}_{p1i}-{R}_{bc}{\dot{\theta }}_{c}-{\dot{e}}_{sp1i}\left(t\right)){R}_{bs}\\ +{C}_{p2ir1}({R}_{br1}{\dot{\theta }}_{r1}-{R}_{bp2i}{\dot{\theta }}_{p2i}-{R}_{bc}{\dot{\theta }}_{c}-{\dot{e}}_{p2ir1}\left(t\right)){R}_{bs})\\ +{C}_{c1}\left({\dot{\theta }}_{c}-{\dot{\theta }}_{c1}\right)+{K}_{c1}\left({\theta }_{c}-{\theta }_{c1}\right)=0\\ {J}_{c1}{\ddot{\theta }}_{c1}-{C}_{c1}\left({\dot{\theta }}_{c}-{\dot{\theta }}_{c1}\right)-{K}_{c1}\left({\theta }_{c}-{\theta }_{c1}\right)=-{T}_{cl}\\ {J}_{c2}{\ddot{\theta }}_{c2}+{C}_{c2}\left({\dot{\theta }}_{c2}-{\dot{\theta }}_{O}\right)+{K}_{c2}\left({\theta }_{c2}-{\theta }_{O}\right)={T}_{cl}\\ {J}_{O}{\ddot{\theta }}_{O}-{C}_{c2}\left({\dot{\theta }}_{c2}-{\dot{\theta }}_{O}\right)-{K}_{c2}\left({\theta }_{c2}-{\theta }_{O}\right)={T}_{L}\end{array}\right. ,$$

where, *K*_*sp*1_ is the average value of time-varying meshing stiffness;* K*_*sp*1r_ is the amplitude of the RTH harmonic;* ϕ*_*sp*1r_ is the phase Angle of the RTH harmonic;* ε*_*sp*1_ is the coincidence degree between the solar gear and the first planetary gear; The first five harmonics can be taken to get a more accurate accuracy, so R = 5.

Assuming that all the gears are unmodified involute spur gears, and ignoring the bending deformation of the input and output shafts, the pure torsional nonlinear mathematical model of the two-speed transmission system as shown in Eq. (5) can be derived by using the concentrated parameter method and Newton's law.

Where *θ*_*s*_*, θ*_*p*1*i*_*, θ*_*p*2*i*_ and *θ*_*r*1_ are respectively the torsional vibration displacements of the sun wheel, the i planetary wheel of the first stage, the i planetary wheel of the second stage and the inner gear ring (i = 1, 2, N). Differentiation concerning time; Input torque *T*_s_(*t*) is the fluctuation value, which can be expressed as *T*_s_(*t*) = *T*_sm_ + *T*_saT_(*t*), where *T*_sm_ is the mean of the torque, *T*_saT_(*t*) is the instantaneous fluctuation value, and can be expressed as *T*_saT_(*t*) = *T*_saT_sin(*ω*_aT_*t* + *ϕ*_aT_); *e*_*sp*1*i*_(*t*) is the static transmission error between the sun wheel and the first stage i planetary wheel, and is related to the manufacture and assembly of gears. It can be regarded as* e*_*sp*1*i*_(*t*) = *ê*sin(*ω*_e_*t* + *ϕ*_e_).* T*_c1_ is the friction torque of the inner gear ring and the planetary frame, which is related to the pressure applied by the friction clutch, the number of friction plates and the friction coefficient of the friction plates, as shown in Eq. (6), where *R*_*o*_ is the radius of the outer circle of the friction plate, and *R*_*i*_ is the radius of the inner circle of the friction plate.* T*_*L*_(t) is the load torque applied on the output shaft of the driveline.

6 $${T}_{cl}=n{\int }_{{R}_{i}}^{{R}_{o}}dT=\frac{2}{3}\mu n{F}_{n}\left(\frac{{R}_{o}^{3}-{R}_{i}^{3}}{{R}_{o}^{2}-{R}_{i}^{2}}\right)$$

Suppose ${\text{x}}_{\text{s}}={\text{R}}_{\text{bs}}\theta_{\text{s}}$*,* ${\text{x}}_{\text{p1i}}={\text{R}}_{\text{bp1i}}\theta_{\text{p1i}}$, ${\text{x}}_{\text{p2i}}={\text{R}}_{\text{bp2i}}\theta_{\text{p2i}}$, ${\text{x}}_{\text{r1}}={\text{R}}_{\text{br1}}\theta_{\text{r1}}$, ${\text{x}}_{\text{c1}}={\text{R}}_{\text{bc}}\theta_{\text{c1}}$, ${\text{x}}_{\text{c2}}={\text{R}}_{\text{bc}}\theta_{\text{c2}}$, ${\text{x}}_{\text{o}}={\text{R}}_{\text{o}}\theta_{\text{o}}$, ${\text{x}}_{\text{c}}={\text{R}}_{\text{bc}}\theta_{\text{c}}$, where ${x}_{s}$, ${x}_{p1i}$, ${x}_{p2i}$, ${x}_{r1}$, ${x}_{c1}$, ${x}_{c2}$, ${x}_{o}$ are the equivalent linear displacements of the solar wheel, the planetary wheel 1, the planetary wheel 2, the inner gear ring, the input end of the friction clutch, the output end of the friction clutch, and the output shaft, respectively. By defining new variables ${\text{X}}_{\text{sp1i}}={\text{x}}_{\text{s}}-{\text{x}}_{\text{p1i}}-{\text{x}}_{\text{c}}-{\text{e}}_{\text{sp1i}}\left({\text{t}}\right)$, ${\text{X}}_{\text{r1p2i}}={\text{x}}_{\text{r1}}-{\text{x}}_{\text{p2i}}-{\text{x}}_{\text{c}}-{\text{e}}_{\text{r1p2i}}\left({\text{t}}\right)$, ${\text{X}}_{\text{p1ip2i}}={\text{x}}_{\text{p1i}}+{\text{x}}_{\text{p2i}}-{\text{e}}_{\text{p1ip2i}}\left({\text{t}}\right)$, ${\text{X}}_{\text{cc1}}={\text{x}}_{\text{c}}-{\text{x}}_{\text{c1}}$, ${\text{X}}_{\text{cl}}={\text{x}}_{\text{c1}}-{\text{x}}_{\text{c2}}$, ${\text{X}}_{\text{c2o}}={\text{x}}_{\text{c2}}-{\text{x}}_{\text{o}}$, ${\text{X}}_{\text{c1}}={\text{x}}_{\text{c1}}$, ${\text{X}}_{\text{c2}}={\text{x}}_{\text{c2}}$, ${\text{X}}_{\text{o}}={\text{x}}_{\text{o}}$.

The dimensionless time scale ${\tau}=t{aa}_{n}$ and the displacement scale ${b}_{c}$ are defined. ${\text{aa}}_{\text{n}}=\sqrt{{\text{K}}_{\text{sp1i}}{(}{\text{r}}_{\text{bs}}^{2}/{\text{J}}_{\text{bs}}+{\text{R}}_{\text{p1i}}^{2}/{\text{J}}_{\text{p1i}}{)}}$, ${\text{X}}_{\text{sp1i}}\text{(t)=}{\text{b}}_{\text{c}}{{\text{q}}}_{\text{1i}}{\tau}$, ${\text{X}}_{\text{r1p2i}}\text{(t)=}{\text{b}}_{\text{c}}{{\text{q}}}_{\text{2i}}{\tau}$, ${\text{X}}_{\text{p1ip2i}}\text{(t)=}{\text{b}}_{\text{c}}{{\text{q}}}_{\text{3i}}{\tau}$, ${\text{X}}_{\text{cc1}}\text{(t)=}{\text{b}}_{\text{c}}{{\text{q}}}_{4}{\tau}$, ${\text{X}}_{\text{cl}}\text{(t)=}{\text{b}}_{\text{c}}{{\text{q}}}_{5}{\tau}$, ${\text{X}}_{\text{c2o}}\text{(t)=}{\text{b}}_{\text{c}}{{\text{q}}}_{6}{\tau}$, ${\text{X}}_{\text{c1}}\text{(t)=}{\text{b}}_{\text{c}}{{\text{q}}}_{7}{\tau}$, ${\text{X}}_{\text{c2}}\text{(t)=}{\text{b}}_{\text{c}}{{\text{q}}}_{8}{\tau}$, ${\text{X}}_{\text{o}}\text{(t)=}{\text{b}}_{\text{c}}{{\text{q}}}_{9}{\tau}$.

7 $$\left[\begin{array}{ccccccccc}1& 0& 0& 0& 0& 0& 0& 0& 0\\ 0& 1& 0& 0& 0& 0& 0& 0& 0\\ 0& 0& 1& 0& 0& 0& 0& 0& 0\\ 0& 0& 0& 1& 0& 0& 0& 0& 0\\ 0& 0& 0& 0& 1& 0& 0& 0& 0\\ 0& 0& 0& 0& 0& 1& 0& 0& 0\\ 0& 0& 0& 0& 0& 0& 1& 0& 0\\ 0& 0& 0& 0& 0& 0& 0& 1& 0\\ 0& 0& 0& 0& 0& 0& 0& 0& 1\end{array}\right]\left[\begin{array}{c}\begin{array}{c}{\ddot{q}}_{1i}\\ {\ddot{q}}_{2i}\\ {\ddot{q}}_{3i}\end{array}\\ \begin{array}{c}{\ddot{q}}_{4}\\ {\ddot{q}}_{5}\\ {\ddot{q}}_{6}\end{array}\\ \begin{array}{c}{\ddot{q}}_{7}\\ {\ddot{q}}_{8}\\ {\ddot{q}}_{9}\end{array}\end{array}\right]+\left[\begin{array}{ccccccccc}{\epsilon }_{11i}& 0& {\epsilon }_{13i}& 0& 0& 0& 0& 0& 0\\ 0& {\epsilon }_{22i}& {\epsilon }_{23i}& 0& 0& 0& 0& 0& 0\\ {\epsilon }_{31i}& {\epsilon }_{32i}& {\epsilon }_{33i}& {\epsilon }_{34i}& 0& 0& 0& 0& 0\\ 0& {\epsilon }_{42}& 0& {\epsilon }_{44}& 0& 0& 0& 0& 0\\ 0& 0& 0& {\epsilon }_{54}& 0& 0& 0& {\epsilon }_{58}& {\epsilon }_{59}\\ 0& 0& 0& 0& 0& 0& 0& {\epsilon }_{68}& {\epsilon }_{69}\\ 0& 0& 0& {\epsilon }_{74}& 0& 0& 0& 0& 0\\ 0& 0& 0& 0& 0& 0& 0& {\epsilon }_{88}& {\epsilon }_{89}\\ 0& 0& 0& 0& 0& 0& 0& {\epsilon }_{98}& {\epsilon }_{99}\end{array}\right]\left[\begin{array}{c}\begin{array}{c}{\dot{q}}_{1i}\\ {\dot{q}}_{2i}\\ {\dot{q}}_{3i}\end{array}\\ \begin{array}{c}{\dot{q}}_{4}\\ {\dot{q}}_{5}\\ {\dot{q}}_{6}\end{array}\\ \begin{array}{c}{\dot{q}}_{7}\\ {\dot{q}}_{8}\\ {\dot{q}}_{9}\end{array}\end{array}\right]+\left[\begin{array}{ccccccccc}{k}_{11i}& 0& {k}_{13i}& 0& 0& 0& 0& 0& 0\\ 0& {k}_{22i}& {k}_{23i}& 0& 0& 0& 0& 0& 0\\ {k}_{31i}& {k}_{32i}& {k}_{33i}& {k}_{34i}& 0& 0& 0& 0& 0\\ 0& {k}_{42}& 0& {k}_{44}& 0& 0& 0& 0& 0\\ 0& 0& 0& {k}_{54}& 0& 0& 0& {k}_{58}& {k}_{59}\\ 0& 0& 0& 0& 0& 0& 0& {k}_{68}& {k}_{69}\\ 0& 0& 0& {k}_{74}& 0& 0& 0& 0& 0\\ 0& 0& 0& 0& 0& 0& 0& {k}_{88}& {k}_{89}\\ 0& 0& 0& 0& 0& 0& 0& {k}_{98}& {k}_{99}\end{array}\right]\left[\begin{array}{c}\begin{array}{c}{q}_{1i}\\ {q}_{2i}\\ {q}_{3i}\end{array}\\ \begin{array}{c}{q}_{4}\\ {q}_{5}\\ {q}_{6}\end{array}\\ \begin{array}{c}{q}_{7}\\ {q}_{8}\\ {q}_{9}\end{array}\end{array}\right]=\left[\begin{array}{c}\begin{array}{c}{P}_{S}-{\ddot{e}}_{sp1i}\left(t\right)\\ {P}_{r1}-{\ddot{e}}_{r1p1i}\left(t\right)\\ -{\ddot{e}}_{p1ip2i}\left(t\right)\end{array}\\ -\begin{array}{c}{P}_{1}\\ {P}_{1}-{P}_{2}\\ {P}_{2}+{P}_{3}\end{array}\\ \begin{array}{c}-{P}_{1}\\ {P}_{2}\\ {P}_{3}\end{array}\end{array}\right]$$

where:

$${\epsilon }_{11i}=\sum_{i=1}^{N}\frac{{C}_{sp1i}}{{m}_{S}{\omega }_{n}}+\frac{{C}_{sp1i}}{{m}_{p1i}{\omega }_{n}}$$, $${\epsilon }_{13i}=-\frac{{C}_{p1ip2i}}{{m}_{p1i}{\omega }_{n}}$$, $${\epsilon }_{22i}=\sum_{i=1}^{N}\frac{{C}_{p2ir1}}{{m}_{r1}{\omega }_{n}}-\frac{{C}_{p2ir1}}{{m}_{p2i}{\omega }_{n}}$$, $${\epsilon }_{23i}=\frac{{C}_{p1ip2i}}{{m}_{p2i}{\omega }_{n}}$$, $${\epsilon }_{31i}=-\frac{{C}_{sp1i}}{{m}_{p1i}{\omega }_{n}}$$, $${\epsilon }_{32i}=\sum_{i=1}^{N}\frac{4{C}_{p2ir1}}{{m}_{c}{\omega }_{n}}+\frac{{C}_{p2ir1}}{{m}_{p2i}{\omega }_{n}}$$, $${\epsilon }_{33i}=\frac{{C}_{p1ip2i}}{{m}_{p1i}{\omega }_{n}}-\frac{{C}_{p1ip2i}}{{m}_{p2i}{\omega }_{n}}$$, $${\epsilon }_{34i}=-\frac{2{C}_{c1}}{{m}_{c}{R}_{bc}^{2}{\omega }_{n}}$$, $${\epsilon }_{42}=-\sum_{i=1}^{N}\frac{2{C}_{p2ir1}}{{m}_{c}{\omega }_{n}}$$, $${\epsilon }_{44}=\frac{{C}_{c1}}{{R}_{bc}^{2}{m}_{c}{\omega }_{n}}+\frac{{C}_{c1}}{{R}_{bc}^{2}{m}_{c1}{\omega }_{n}}$$, $${\epsilon }_{54}=-\frac{{C}_{c1}}{{R}_{bc}^{2}{m}_{c1}{\omega }_{n}}$$, $${\epsilon }_{58}=-\frac{{C}_{c2}}{{R}_{bc}^{2}{m}_{c2}{\omega }_{n}}$$, $${\epsilon }_{59}=\frac{{C}_{c2}}{{R}_{bc}{{R}_{O}m}_{c2}{\omega }_{n}}$$, $${\epsilon }_{68}=\frac{{C}_{c2}}{{R}_{bc}^{2}{m}_{c2}{\omega }_{n}}+\frac{{C}_{c2}}{{R}_{bc}^{2}{m}_{O}{\omega }_{n}}$$, $${\epsilon }_{69}=-\frac{{C}_{c2}}{{R}_{bc}{{R}_{O}m}_{c2}{\omega }_{n}}-\frac{{C}_{c2}}{{R}_{bc}{{R}_{O}m}_{O}{\omega }_{n}}$$, $${\epsilon }_{88}=\frac{{C}_{c2}}{{R}_{bc}^{2}{m}_{c2}{\omega }_{n}}$$, $${\epsilon }_{89}=-\frac{{C}_{c2}}{{R}_{bc}{{R}_{O}m}_{c2}{\omega }_{n}}$$, $${\epsilon }_{98}=-\frac{{C}_{c2}}{{R}_{bc}^{2}{m}_{O}{\omega }_{n}}$$, $${\epsilon }_{99}=\frac{{C}_{c2}}{{R}_{bc}{{R}_{O}m}_{O}{\omega }_{n}}$$, $${k}_{11i}=\sum_{i=1}^{N}\frac{{K}_{sp1i}}{{m}_{S}{{\omega }_{n}}^{2}}+\frac{{K}_{sp1i}}{{m}_{p1i}{{\omega }_{n}}^{2}}$$, $${k}_{13i}=-\frac{{K}_{p1ip2i}}{{m}_{p1i}{{\omega }_{n}}^{2}}$$, $${k}_{22i}=\sum_{i=1}^{N}\frac{{K}_{p2ir1}}{{m}_{r1}{{\omega }_{n}}^{2}}-\frac{{K}_{p2ir1}}{{m}_{p2i}{{\omega }_{n}}^{2}}$$, $${k}_{23i}=\frac{{K}_{p1ip2i}}{{m}_{p2i}{{\omega }_{n}}^{2}}$$, $${k}_{31i}=-{K}_{12i}=\frac{{K}_{sp1i}}{{m}_{p1i}{{\omega }_{n}}^{2}}$$, $${k}_{32i}=\frac{{K}_{p2ir1}}{{m}_{p2i}{{\omega }_{n}}^{2}}+\sum_{i=1}^{N}\frac{4{K}_{p2ir1}}{{m}_{c}{{\omega }_{n}}^{2}}$$, $${k}_{33i}=\frac{{K}_{p1ip2i}}{{m}_{p1i}{{\omega }_{n}}^{2}}-\frac{{K}_{p1ip2i}}{{m}_{p2i}{{\omega }_{n}}^{2}}$$, $${k}_{34i}=-\frac{2{K}_{c1}}{{m}_{c}{R}_{bc}^{2}{{\omega }_{n}}^{2}}$$, $${k}_{42}=-\sum_{i=1}^{N}\frac{2{K}_{p2ir1}}{{m}_{c}{{\omega }_{n}}^{2}}$$, $${k}_{44}=\frac{{K}_{c1}}{{R}_{bc}^{2}{m}_{c}{{\omega }_{n}}^{2}}+\frac{{K}_{c1}}{{R}_{bc}^{2}{m}_{c1}{{\omega }_{n}}^{2}}$$, $${k}_{54}=-\frac{{K}_{c1}}{{R}_{bc}^{2}{m}_{c1}{{\omega }_{n}}^{2}}$$, $${k}_{58}=-\frac{{K}_{c2}}{{R}_{bc}^{2}{m}_{c2}{{\omega }_{n}}^{2}}$$,$${k}_{59}=\frac{{K}_{c2}}{{R}_{bc}{{R}_{O}m}_{c2}{{\omega }_{n}}^{2}}$$, $${k}_{68}=\frac{{K}_{c2}}{{R}_{bc}^{2}{m}_{c2}{{\omega }_{n}}^{2}}+\frac{{K}_{c2}}{{R}_{bc}^{2}{m}_{O}{{\omega }_{n}}^{2}}$$, $${k}_{69}=-\frac{{K}_{c2}}{{R}_{bc}{{R}_{O}m}_{c2}{{\omega }_{n}}^{2}}-\frac{{K}_{c2}}{{R}_{bc}{{R}_{O}m}_{O}{{\omega }_{n}}^{2}}$$, $${k}_{88}=\frac{{K}_{c2}}{{R}_{bc}^{2}{m}_{c2}{{\omega }_{n}}^{2}}$$, $${k}_{89}=-\frac{{K}_{c2}}{{R}_{bc}{{R}_{O}m}_{c2}{{\omega }_{n}}^{2}}$$, $${k}_{98}=\frac{{K}_{c2}}{{R}_{bc}^{2}{m}_{O}{{\omega }_{n}}^{2}}$$, $${k}_{99}=-\frac{{K}_{c2}}{{R}_{bc}{{R}_{O}m}_{O}{{\omega }_{n}}^{2}}$$, $${P}_{S}=\frac{{F}_{S}}{{m}_{S}{b}_{c}{\omega }_{n}^{2}}$$, $${P}_{r1}=\frac{{F}_{r1}}{{m}_{r1}{b}_{c}{\omega }_{n}^{2}}$$, $${P}_{1}=\frac{{F}_{cl}}{{m}_{c1}{b}_{c}{\omega }_{n}^{2}}$$, $${P}_{2}=\frac{{F}_{cl}}{{m}_{c2}{b}_{c}{\omega }_{n}^{2}}$$, $${P}_{3}=\frac{{F}_{L}}{{m}_{O}{b}_{c}{\omega }_{n}^{2}}$$.

Including $${m}_{s}=\frac{{J}_{s}}{{{r}_{bs}}^{2}}$$, $${m}_{p1i}=\frac{{J}_{p1i}}{{{r}_{bp1i}}^{2}}$$, $${m}_{p2i}=\frac{{J}_{p2i}}{{{r}_{bp2i}}^{2}}$$, $${m}_{r}=\frac{{J}_{r}}{{{r}_{br}}^{2}}$$, $${m}_{c}=\frac{{J}_{c}}{{{r}_{bc}}^{2}}$$.

## Parameter study

The basic parameters of the two-speed transmission system are shown in Table 1. The Runge–Kutta method with fourth-order variable step size is used to solve the equation set 7. The initial values of all displacements are 0, and the initial values of all velocities are 0.01, and the solution interval is [0, 600t].

**Table 1:** Two-speed transmission system parameters.

Name of parameter	Parameters of the code	Parameter values
Output shaft radius	*R*_*o*_	0.0260 m
Output shaft moment of inertia	*J*_*o*_	0.19738 kg m^2^
The moment of inertia of the solar wheel	*J*_*s*_	0.0534 kg m^2^
The moment of inertia of planetary wheel I of the first stage	*J*_*p*1*i*_	0.000235 kg m^2^
The moment of inertia of the second stage I planet wheel Moment of inertia of inner ring gear	*J*_*p*2*i*_	0.000153 kg m^2^
The torsional stiffness of the sun wheel and the first stage I planet wheel	*J*_*r*1_	0.3793 kg m^2^
Torsional stiffness of second class I planetary gear and inner gear ring	*K*_*sp*1*i*_	6.8655 × 10^8^ N m/rad
Torsional stiffness of planetary frame and friction clutch input end	*K*_*p*1*ip*2*i*_	2.2356 × 10^8^ N m/rad
Torsional stiffness of output end and output shaft of friction clutch	*K*_*p*2*ir*1_	2.6551 × 10^8^ N m/rad
The sun wheel is damped by meshing with the first stage I planet wheel	*K*_*c*1_	1.32 × 10^5^ N m/rad
The first planetary wheel engages with the second planetary wheel for damping	*K*_*c*2_	1.32 × 10^5^ N m/rad
The second stage planetary gear is damped by meshing with the inner gear ring	*C*_*sp*1*i*_	2.4379 × 10^3^
Planetary frame with torsional damping of friction clutch input end	*C*_*p*1*ip*2*i*_	939.31
Friction clutch output end with torsional damping output shaft	*C*_*p*2*ir*1_	1.3628 × 10^3^
Torsional stiffness of second class I planetary gear and inner gear ring	*C*_*c*1_	50
Torsional stiffness of planetary frame and friction clutch input end	*C*_*c*2_	50
Number of solar gear	*Z*_*s*_	54
Number of first planetary gear teeth	*Z*_*p*1*i*_	21
Number of second planetary gear	*Z*_*p*2*i*_	19
Number of inner ring teeth	*Z*_*r*1_	108
Degree of contact between the sun wheel and the first planetary wheel	${\epsilon }_{sp1i}$	1.6684
Degree of convergence between the first and second planetary wheels	${\epsilon }_{p1ip2i}$	1.5564
Second planetary wheel and inner gear ring coincidence degree	${\epsilon }_{p2ir1}$	1.8154
Solar wheel input force	${F}_{S}$	3.6959 × 10^4^ *N*
Internal gear ring input force	${F}_{r1}$	1.8480 × 10^4^ *N*
load	${F}_{L}$	54,881 *N*
Friction clutch output force	${F}_{cl}$	5936 *N*

### The influence of excitation frequency

**Figure 3:**
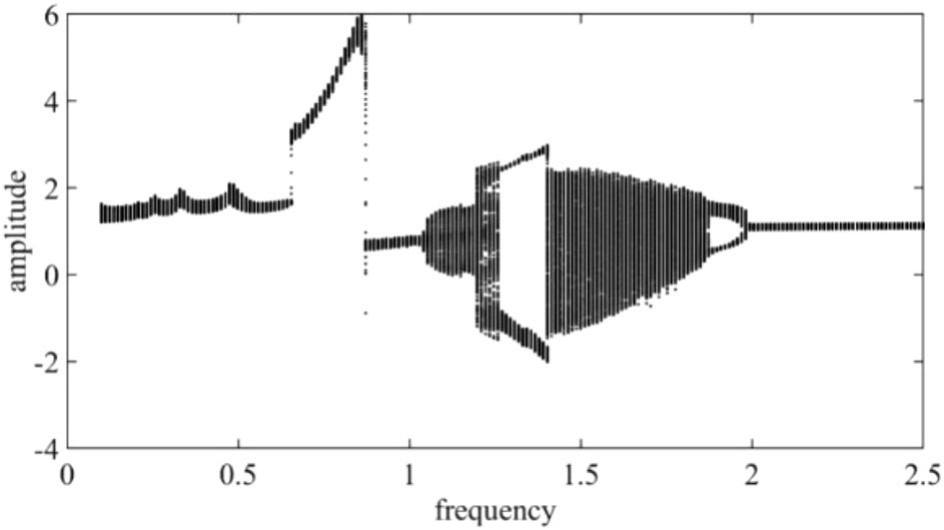
Global bifurcation diagram of torsional vibration of internal gear ring varying with excitation frequency ωh.

**Figure 4:**
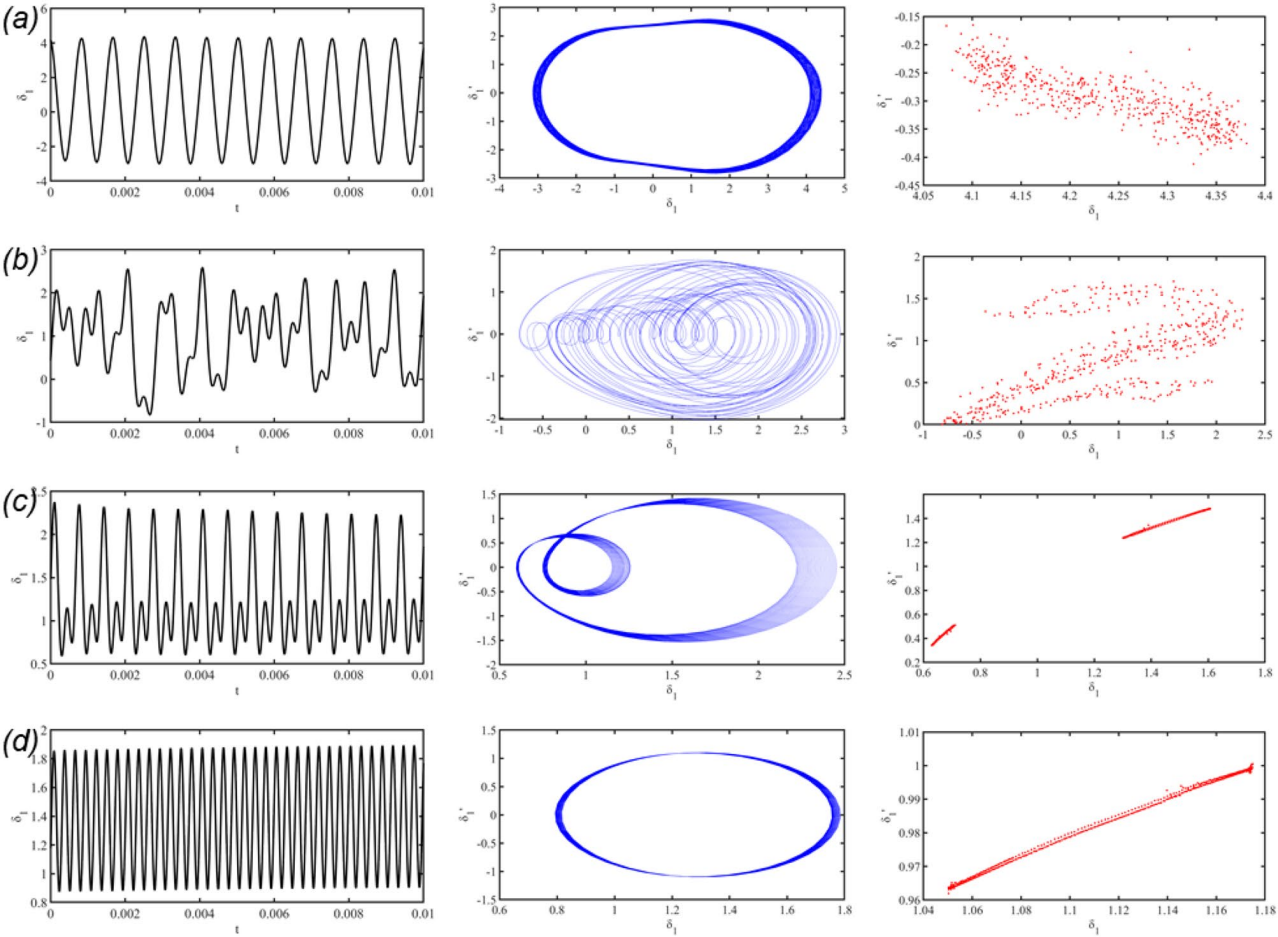
Time domain diagram, phase diagram and Poincare cross section diagram corresponding to different excitation frequency points of inner gear ring: (**a**) ωh = 0.7633, (**b**) ωh = 1.6196, (**c**) ωh = 1.9332, (**d**) ωh = 2.2588.

Figure 3 shows the dimensionless torsional vibration bifurcation diagram of the inner gear ring drawn with the excitation frequency of ωh as the control variable in the high speed mode of the two-speed transmission system. It can be seen from the Fig. 3 that there is an obvious jump phenomenon at ωh = 0.65 and at ωh = 0.87 of the inner gear ring. When 0.65 ≤  ωh ≤ 0.87, the inner gear ring moves in a single periodic motion, and the vibration increases with the increase of the excitation frequency. When the excitation frequency is in the low frequency band (ωh ≤ 0.65 and 0.87 ≤ ωh ≤ 1.02) and the high frequency band (ωh ≥ 2.0), the torsional vibration is a relatively stable single periodic motion, which is not affected by the excitation frequency. When the excitation frequency is in the range of 1.04 ≤ ωh ≤ 1.25, 1.41 ≤ ωh ≤ 1.87, the inner gear ring enters the chaotic region. When the excitation frequency is within 1.27 ≤ ωh ≤ 1.4, the motion of the system is a double cycle motion. In the range of 1.88 ≤ ωh ≤ 1.98, the movement of the system changes from double period to single period. Figure 4 shows the time domain diagram, phase diagram and Poincare cross section diagram of the system at the excitation frequency point ωh = 0.7633, 1.6196, 1.9332 and 2.2588, which respectively correspond to quasi-periodic, chaotic, double cycle and haploid periodic motions.

### Influence law of dynamic friction coefficient of friction clutch

**Figure 5:**
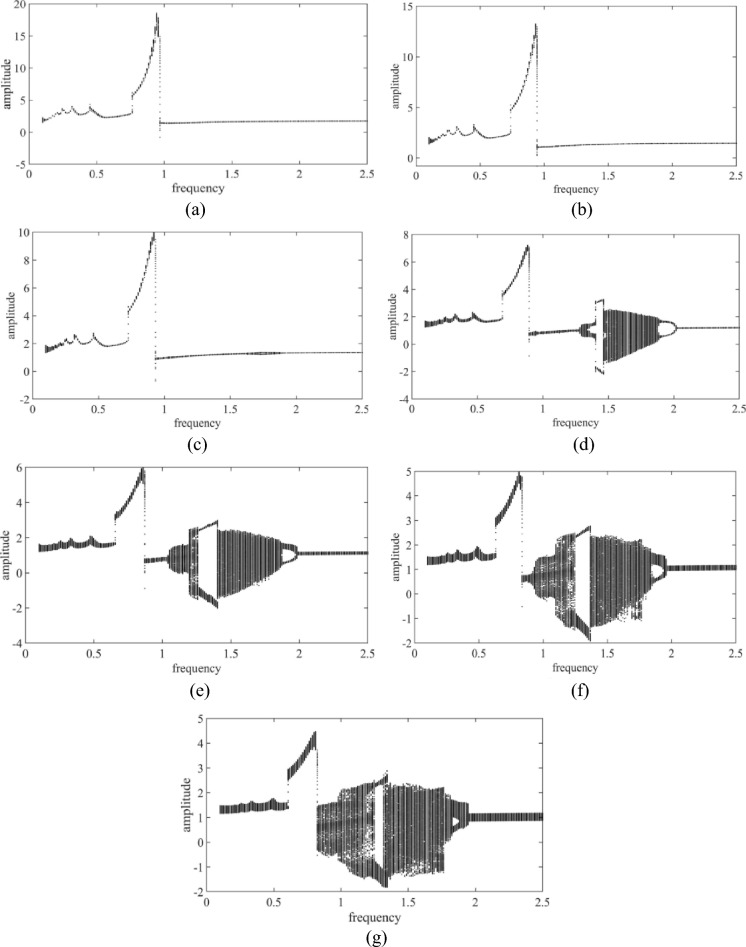
Influence of dynamic friction coefficient of friction clutch on bifurcation characteristics of internal gear ring. (**a**) μ = 0.17, (**b**) μ = 0.16, (**c**) μ = 0.15, (**d**) μ = 0.14, (**e**) μ = 0.13, (**f**) μ = 0.12, (**g**) μ = 0.11.

Figure 5 shows the influence of dynamic friction coefficient of friction clutch on bifurcation characteristics of internal gear ring. As can be seen from the figure, with the increase of the dynamic friction coefficient, the oscillation value of the system in the unstable region can be effectively suppressed. From Fig. 5a–c, it can be seen that the system experienced quasi-periodic motion and haploperiodic motion in the whole excitation frequency range. In the low frequency band ωh ≤ 0.5, the motion of the system fluctuates up and down in a small range, and enters a quasi-periodic motion in the range of 0.5 ≤ωh ≤ 0.6, and then appears an obvious jump phenomenon. When the excitation frequency ωh ≥ 1.0, the dynamic friction coefficient of the friction clutch has almost no effect on the bifurcation characteristics of the system. With the increase of the excitation frequency, the system keeps a single periodic motion. As can be seen from Fig. 5d–g, the bifurcation structure, motion form and chaotic response region of the system are significantly changed when the dynamic friction coefficient is small. In the range of low frequency band (ωh ≤ 0.65), with the reduction of friction coefficient, the fluctuation caused by the system movement is effectively alleviated. As the excitation frequency increases, it can be seen from the Fig. 5 that there is an obvious jump phenomenon, but the peak of the jump can be reduced by reducing the friction coefficient. When the excitation frequency ωh ≥ 0.9, the system experienced a single periodic motion, chaotic motion, and double periodic motion. When the excitation frequency is 0.9 ≤ ωh ≤ 1.5, the movement of the system enters into chaotic motion from single-fold periodic motion and then into double periodic motion. In this frequency range, with the decrease of friction coefficient, the area of chaotic response of the system gradually widens and the single-fold periodic motion is reduced. This shows that the reduction of the dynamic friction coefficient of the friction clutch makes the motion response of the system more complex in the excitation frequency of 0.9 ≤ ωh ≤ 1.5. In the range of high frequency band (ωh ≥ 2.0), the dynamic friction coefficient of friction clutch has almost no effect on the bifurcation structure and motion form of the system, and the system keeps the single-fold periodic motion.

## Conclusion

The dynamic differential equation of pure torsional nonlinear dynamic load is established by lumped parameter method, and the parameters of planetary wheel number, backlash and clutch dynamic load are considered in the modeling process, and the equations are dimensionless. Then the fourth order Runge–Kutta method with variable step size is used to solve the dimensionless differential equation, and the phase diagram and Poincare diagram in the high-speed gear file are obtained. By changing the friction coefficient of the friction clutch, the influence of parameter change on the nonlinear dynamic characteristics of the system is analyzed, and the following conclusions are obtained:

1. With the increase of excitation frequency, the system went through a single period, a quasi period, chaos, a double period, and then the second period turned into chaotic motion, and then the second period turned into a double period and a single period motion, and the path to the chaos was found out. To make the system have good dynamic characteristics, the excitation frequency of the system should be guaranteed to be small or large.
2. In the low-frequency band (ωh ≤ 0.9), the friction coefficient of the friction clutch can be reduced to reduce the vibration amplitude; In the middle-frequency band (0.9 ≤ ωh ≤ 1.9), reducing the friction coefficient will make the system tend to be unstable vibration. In the high-frequency band (1.9 ≤ ωh ≤ 2.5), it is a single-times periodic motion, which is not affected by the friction coefficient. In order to make the system have good stability and reduce the vibration of the system during operation, when the system is in the low-frequency stage, it is more appropriate to select a smaller value for the friction coefficient of the friction clutch; When the system is in the intermediate frequency stage, it is more appropriate to take a larger value for the friction coefficient of the friction clutch; When the system is in the high-frequency phase, the friction coefficient can be optionally selected because the coefficient of friction has little effect on the stability of the system.

## Limitations and deficiencies

1. This paper adopts the lumped parameter method in modeling and the Runge–Kutta method in solving differential equations, which is a commonly used modeling and solving method for system dynamics. However, the lumped parameter method is a simplified modeling method, which usually regards the shaft as a point mass or a rigid connection, and has certain limitations in capturing the deflection and deformation of the shaft.
2. In future studies, under the condition that the amount of calculation is appropriate, if the accuracy is required to be high, the potential energy method or Timoshenko theory can be considered for modeling, which will make the results more accurate.

## Data Availability

All data, models, and codes generated or used during the study are included within the article.
